# Supplementary material for: sciMET-cap: high-throughput single-cell methylation analysis with a reduced sequencing burden
Source: Genome Biol. 2024 Jul 10;25:186. doi: 10.1186/s13059-024-03306-7 (PMC11234687; doi:10.1186/s13059-024-03306-7)
Supplement: Supplementary file 1 — Additional file 1. Supplementary Figures S1-S5. [file 13059_2024_3306_MOESM1_ESM.pdf]

sciMET-cap: high-throughput single-cell methylation analysis with a reduced sequencing burden

Sonia N. Acharya[1,a], Ruth V. Nichols[1,a], Lauren E. Rylaarsdam[1], Brendan L. O'Connell[1,2], Theodore P. Braun[2,3,4], Andrew C. Adey[1,2,3,5]*

1. Department of Molecular & Medical Genetics, Oregon Health & Science University, Portland, OR, USA
2. Cancer Early Detection Advanced Research Center, Oregon Health and Science University, Portland, OR, USA
3. Knight Cancer Institute, Oregon Health and Science University, Portland, OR, USA
4. Division of Hematology/Medical Oncology, School of Medicine, Oregon Health & Science University, Portland, OR, USA
5. Knight Cardiovascular Institute, Oregon Health and Science University, Portland, OR, USA

[a] These authors contributed equally to this work
* Correspondence: adey@ohsu.edu

Additional File 1: Figures S1-5

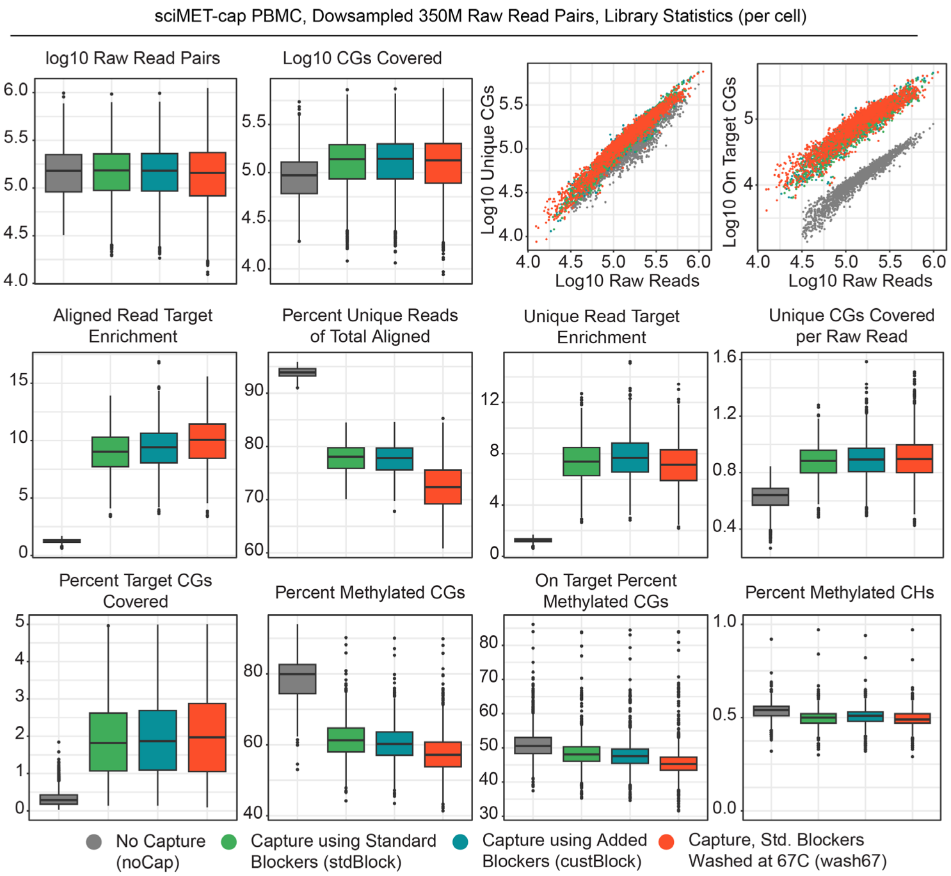

Figure S1. sciMET-cap comparisons. PBMC sciMETv2 and sciMET-cap datasets for four conditions at matched read depth are shown across multiple quality metrics and measurements of sequencing efficiency.

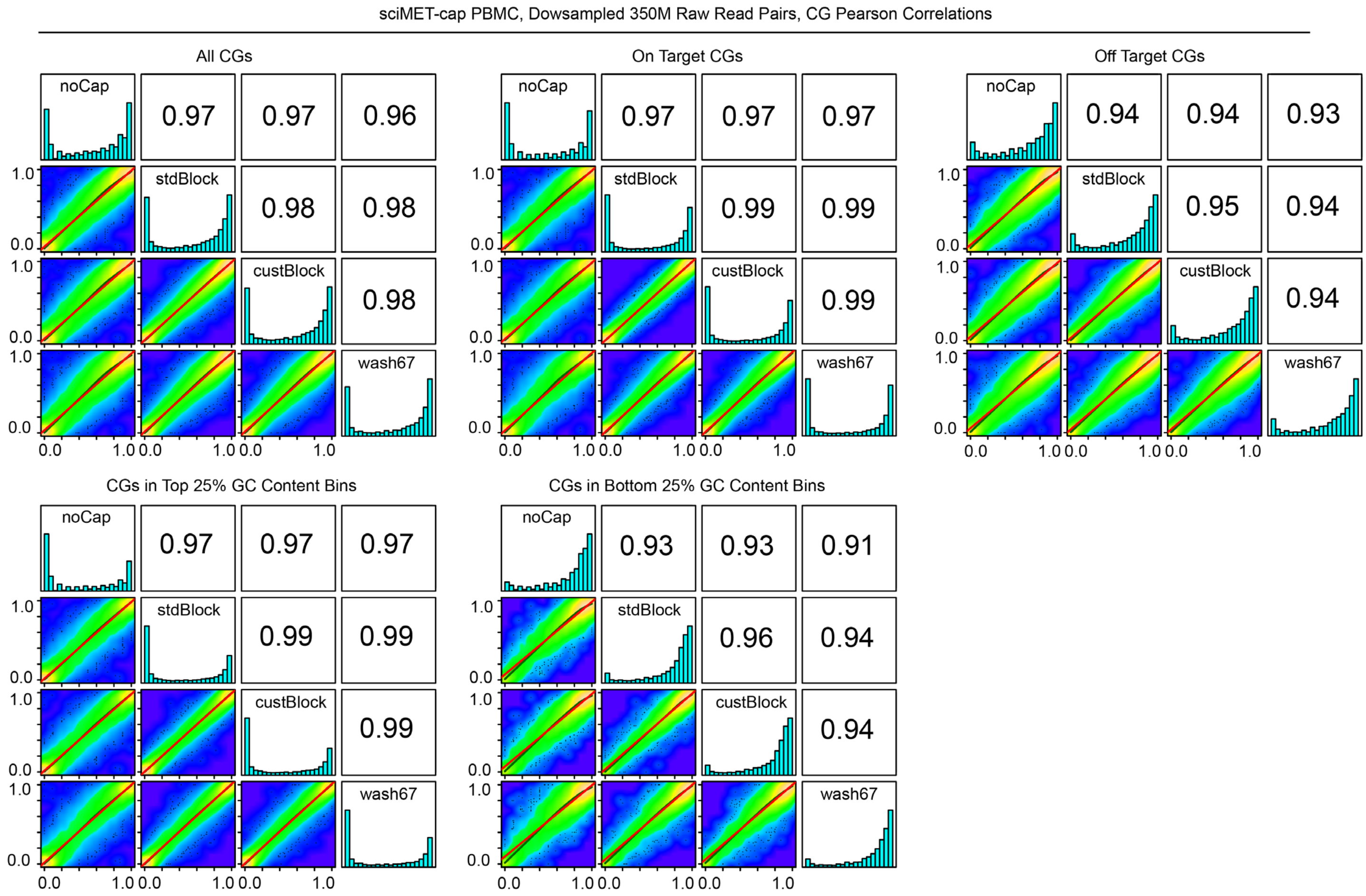

Figure S2. sciMET-cap CG site-level methylation correlations. Pearson correlations between all four conditions for all sites, sites that are either on or off the target capture regions, and sites that are in high or low GC content regions as assessed to be the top or bottom 25% of 2 kbp windows.

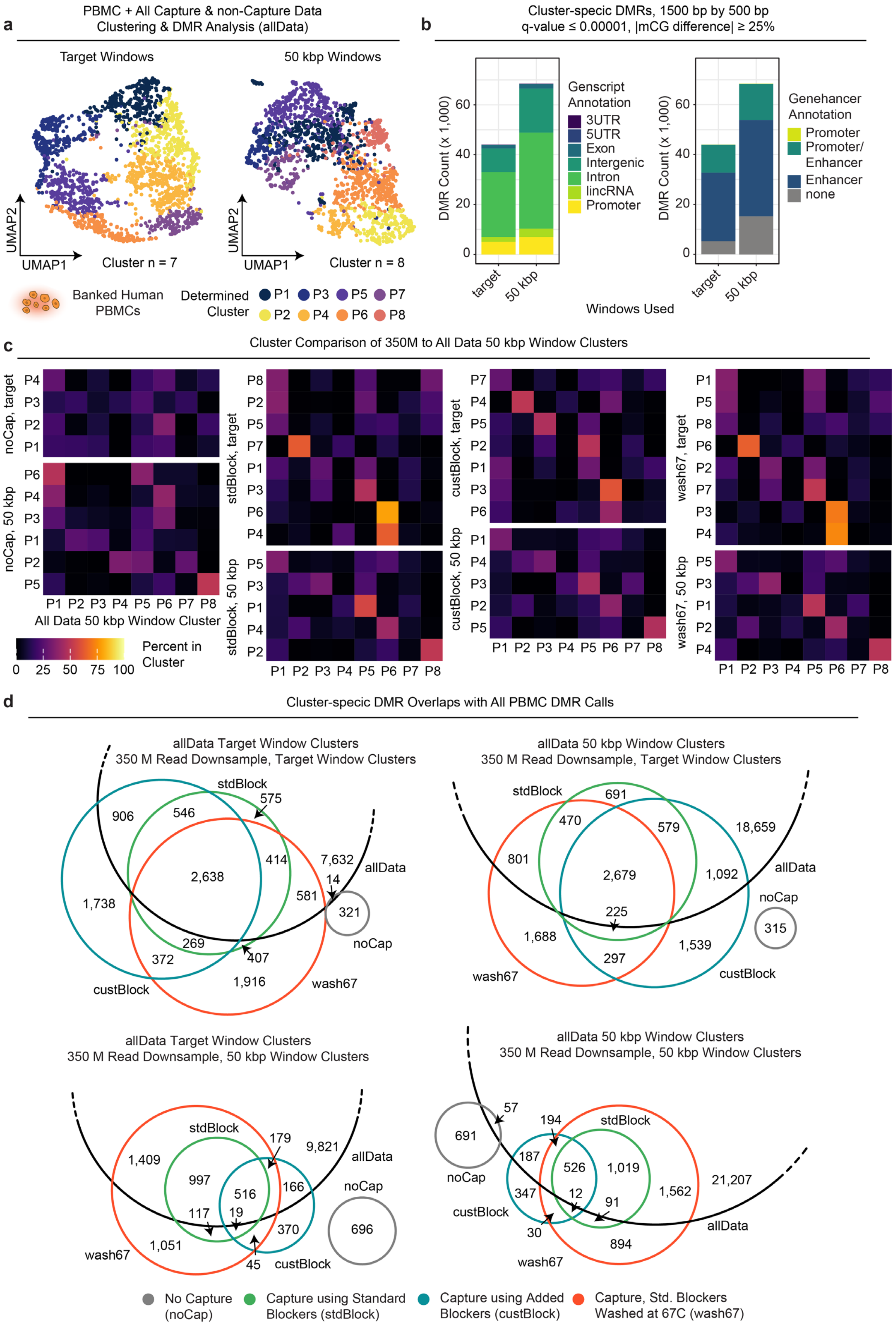
a
PBMC + All Capture & non-Capture Data
Clustering & DMR Analysis (allData)
Target Windows
50 kbp Windows
UMAP1
UMAP2
Cluster n = 7
Cluster n = 8
Banked Human PBMCs
Determined Cluster
P1 P3 P5 P7
P2 P4 P6 P8
b
Cluster-specic DMRs, 1500 bp by 500 bp
q-value ≤ 0.00001, |mCG difference| ≥ 25%
DMR Count (x 1,000)
Genscript Annotation
3UTR
5UTR
Exon
Intergenic
Intron
lincRNA
Promoter
Genehancer Annotation
Promoter
Promoter/ Enhancer
Enhancer
none
target
50 kbp
Windows Used
c
Cluster Comparison of 350M to All Data 50 kbp Window Clusters
noCap, target
noCap, 50 kbp
stdBlock, target
stdBlock, 50 kbp
custBlock, target
custBlock, 50 kbp
wash67, target
wash67, 50 kbp
All Data 50 kbp Window Cluster
Percent in Cluster
0 25 50 75 100
d
Cluster-specic DMR Overlaps with All PBMC DMR Calls
allData Target Window Clusters
350 M Read Downsample, Target Window Clusters
allData 50 kbp Window Clusters
350 M Read Downsample, Target Window Clusters
allData Target Window Clusters
350 M Read Downsample, 50 kbp Window Clusters
allData 50 kbp Window Clusters
350 M Read Downsample, 50 kbp Window Clusters
stdBlock
custBlock
wash67
noCap
allData
No Capture (noCap)
Capture using Standard Blockers (stdBlock)
Capture using Added Blockers (custBlock)
Capture, Std. Blockers Washed at 67C (wash67)

Figure S3. Combined PBMC data and comparisons. a. UMAP projections colored by cluster for merged data from all capture and non-capture sequencing runs and conditions using either target windows of 50 kbp tiling windows. b. Cluster-specific DMRs identified in the merged dataset with Genscript and Genehancer annotations. c. Confusion matrixes of cell assignment across clusters for the 350 million read downsampled conditions compared to the aggregated dataset clusters. d. Intersection of cluster-specific DMRs identified across conditions compared with the aggregated dataset DMR calls.

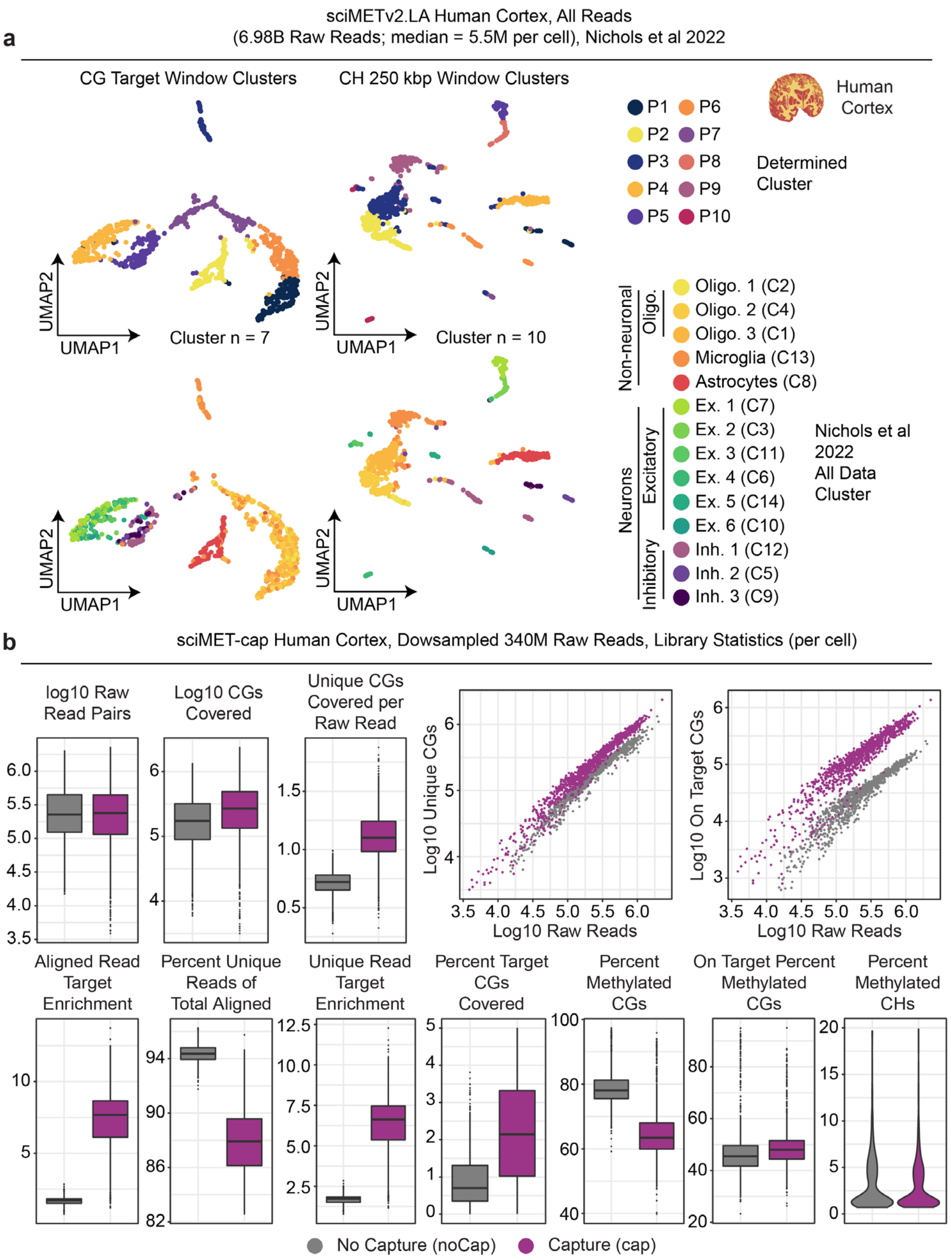

Figure S4. Analysis of sciMETv2 and sciMET-cap on human cortex. a. UMAP projections colored by cluster (top) or previously-determined cell type (bottom) for the roughly 7 billion sequence read sciMETv2 dataset. b. Comparison of sciMETv2 and sciMET-cap across various quality and sequencing efficiency metrics on matched read count data.

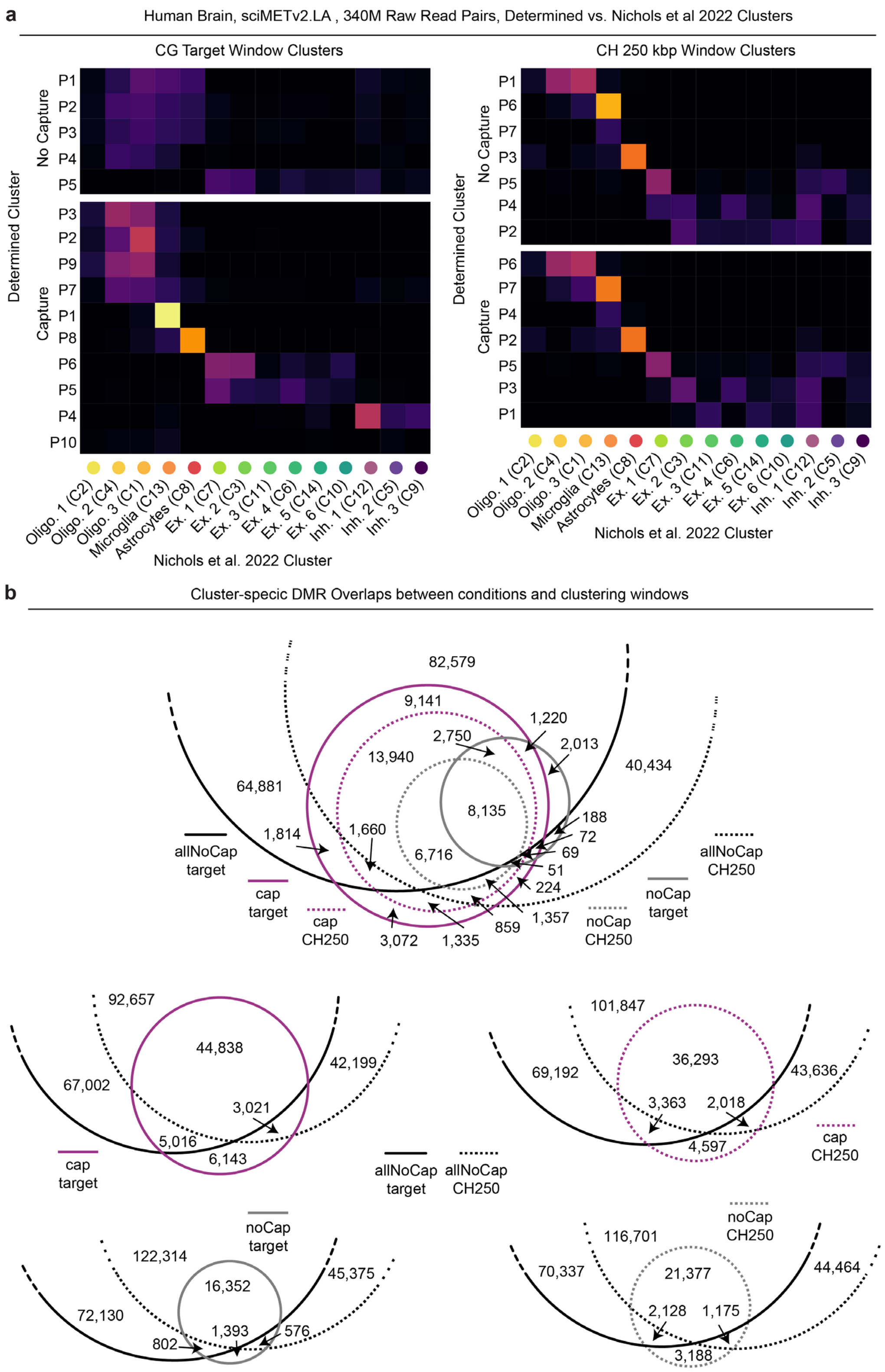

Figure S5. Human brain cluster and DMR comparisons. a. Confusion matrixes of called clusters compared to the previously-defined cell types from Nichols et. al. 2022. b. Intersection of cluster-specific DMRs identified across conditions compared with the high-coverage non-capture dataset (~7 billion reads).
