# Supplementary material for: sciMET-cap: high-throughput single-cell methylation analysis with a reduced sequencing burden
Source: Genome Biol. 2024 Jul 10;25:186. doi: 10.1186/s13059-024-03306-7 (PMC11234687; doi:10.1186/s13059-024-03306-7)
Supplement: Supplementary file 2 — Additional file 2. Detailed statistics and processing of sciMET-cap technical performance data. [file 13059_2024_3306_MOESM2_ESM.html]

sciMET\_CAP\_CellInfo


# sciMET\_CAP\_CellInfo

move to dir, load dependencies & load data

```
setwd("E:\\Data/sciMET_CAP/")
library(ggplot2)
```

```
## Warning: package 'ggplot2' was built under R version 4.1.3
```

```
CellInfo<-read.table("CellInfo.txt", header = TRUE)
rownames(CellInfo) <- CellInfo$cellID
CellInfo<-CellInfo[,-1]
```

define colors

```
noCap="gray50"
noCap.on = "gray30"
custBlock="#089099"
custBlock.on = "#045275"
stdBlock = "#40AD5A"
stdBlock.on = "#06592A"
wash67 = "#FC4E2A"
wash67.on = "#B10026"
cap="#9C3587"
```

plots

```
totalCG_by_raw<-ggplot(data = CellInfo) + theme_bw() +
  geom_point(aes(log10(noCap.raw_reads),log10(noCap.unique_CGs)),color=noCap,size=1) +
  geom_point(aes(log10(custBlock.raw_reads),log10(custBlock.unique_CGs)),color=custBlock,size=1) +
  geom_point(aes(log10(stdBlock.raw_reads),log10(stdBlock.unique_CGs)),color=stdBlock,size=1) +
  geom_point(aes(log10(wash67.raw_reads),log10(wash67.unique_CGs)),color=wash67,size=1) +
  xlab("Log10 Raw Reads per Cell") + ylab(" Log10 Unique CGs per Cell")
totalCG_by_raw
```

```
ggsave(useDingbats=FALSE,filename = "totalCG_by_raw.pdf",plot=totalCG_by_raw,width=4,height=4)

onCG_by_raw<-ggplot(data = CellInfo) + theme_bw() +
  geom_point(aes(log10(noCap.raw_reads),log10(noCap.unique_CG_onTarget)),color=noCap,size=1) +
  geom_point(aes(log10(custBlock.raw_reads),log10(custBlock.unique_CG_onTarget)),color=custBlock,size=1) +
  geom_point(aes(log10(stdBlock.raw_reads),log10(stdBlock.unique_CG_onTarget)),color=stdBlock,size=1) +
  geom_point(aes(log10(wash67.raw_reads),log10(wash67.unique_CG_onTarget)),color=wash67,size=1) +
  xlab("Log10 Raw Reads per Cell") + ylab("Log10 On Target CGs per Cell")
onCG_by_raw
```

```
ggsave(useDingbats=FALSE,filename = "onCG_by_raw.pdf",plot=onCG_by_raw,width=4,height=4)

log10_raw<-ggplot(data = CellInfo) + theme_bw() +
  geom_boxplot(aes(x="1",log10(noCap.raw_reads)),fill=noCap) +
  geom_boxplot(aes(x="2",log10(stdBlock.raw_reads)),fill=stdBlock) +
  geom_boxplot(aes(x="3",log10(custBlock.raw_reads)),fill=custBlock) +
  geom_boxplot(aes(x="4",log10(wash67.raw_reads)),fill=wash67) +
  xlab("Condition") + ylab("log10 Raw Reads per Cell")
log10_raw
```

```
ggsave(useDingbats=FALSE,filename = "log10_raw.pdf",plot=log10_raw,width=4,height=4)

total_CGs<-ggplot(data = CellInfo) + theme_bw() +
  geom_boxplot(aes(x="1",log10(noCap.unique_CGs)),fill=noCap) +
  geom_boxplot(aes(x="2",log10(stdBlock.unique_CGs)),fill=stdBlock) +
  geom_boxplot(aes(x="3",log10(custBlock.unique_CGs)),fill=custBlock) +
  geom_boxplot(aes(x="4",log10(wash67.unique_CGs)),fill=wash67) +
  xlab("Condition") + ylab("Log10 CGs Covered per Cell")
total_CGs
```

```
ggsave(useDingbats=FALSE,filename = "total_CGs.pdf",plot=total_CGs,width=4,height=4)

fold_CG_over_noCap<-ggplot(data = CellInfo) + theme_bw() +
  geom_boxplot(aes(x="2",(stdBlock.unique_CGs/noCap.unique_CGs)),fill=stdBlock) +
  geom_boxplot(aes(x="3",(custBlock.unique_CGs/noCap.unique_CGs)),fill=custBlock) +
  geom_boxplot(aes(x="4",(wash67.unique_CGs/noCap.unique_CGs)),fill=wash67) +
  xlab("Condition") + ylab("CGs Covered over No Capture per Cell")
fold_CG_over_noCap
```

```
ggsave(useDingbats=FALSE,filename = "fold_CG_over_noCap.pdf",plot=fold_CG_over_noCap,width=4,height=4)

pct_CG_covered<-ggplot(data = CellInfo) + theme_bw() +
  geom_boxplot(aes(x="1",noCap.target_CG_covPct),fill=noCap) +
  geom_boxplot(aes(x="2",stdBlock.target_CG_covPct),fill=stdBlock) +
  geom_boxplot(aes(x="3",custBlock.target_CG_covPct),fill=custBlock) +
  geom_boxplot(aes(x="4",wash67.target_CG_covPct),fill=wash67) +
  xlab("Condition") + ylab("Percent Target CGs Covered per Cell") +
  ylim(c(0,5))
pct_CG_covered
```

```
## Warning: Removed 47 rows containing non-finite values (`stat_boxplot()`).
```

```
## Warning: Removed 60 rows containing non-finite values (`stat_boxplot()`).
```

```
## Warning: Removed 71 rows containing non-finite values (`stat_boxplot()`).
```

```
ggsave(useDingbats=FALSE,filename = "pct_CG_covered.pdf",plot=pct_CG_covered,width=4,height=4)
```

```
## Warning: Removed 47 rows containing non-finite values (`stat_boxplot()`).
```

```
## Warning: Removed 60 rows containing non-finite values (`stat_boxplot()`).
```

```
## Warning: Removed 71 rows containing non-finite values (`stat_boxplot()`).
```

```
aligned_enrichment<-ggplot(data = CellInfo) + theme_bw() +
  geom_boxplot(aes(x="1",noCap.aligned_enrichment),fill=noCap) +
  geom_boxplot(aes(x="2",stdBlock.aligned_enrichment),fill=stdBlock) +
  geom_boxplot(aes(x="3",custBlock.aligned_enrichment),fill=custBlock) +
  geom_boxplot(aes(x="4",wash67.aligned_enrichment),fill=wash67) +
  xlab("Condition") + ylab("Aligned Read Target Enrichment per Cell")
aligned_enrichment
```

```
ggsave(useDingbats=FALSE,filename = "aligned_enrichment.pdf",plot=aligned_enrichment,width=4,height=4)

unique_enrichment<-ggplot(data = CellInfo) + theme_bw() +
  geom_boxplot(aes(x="1",noCap.unique_enrichment),fill=noCap) +
  geom_boxplot(aes(x="2",stdBlock.unique_enrichment),fill=stdBlock) +
  geom_boxplot(aes(x="3",custBlock.unique_enrichment),fill=custBlock) +
  geom_boxplot(aes(x="4",wash67.unique_enrichment),fill=wash67) +
  xlab("Condition") + ylab("Unique Read Target Enrichment per Cell")
unique_enrichment
```

```
ggsave(useDingbats=FALSE,filename = "unique_enrichment.pdf",plot=unique_enrichment,width=4,height=4)

pct_unique_reads<-ggplot(data = CellInfo) + theme_bw() +
  geom_boxplot(aes(x="1",((noCap.unique_reads/noCap.aligned_reads)*100)),fill=noCap) +
  geom_boxplot(aes(x="2",((stdBlock.unique_reads/stdBlock.aligned_reads)*100)),fill=stdBlock) +
  geom_boxplot(aes(x="3",((custBlock.unique_reads/custBlock.aligned_reads)*100)),fill=custBlock) +
  geom_boxplot(aes(x="4",((wash67.unique_reads/wash67.aligned_reads)*100)),fill=wash67) +
  xlab("Condition") + ylab("Percent Unique Reads of Total Aligned per Cell")
pct_unique_reads
```

```
ggsave(useDingbats=FALSE,filename = "pct_unique_reads.pdf",plot=pct_unique_reads,width=4,height=4)

CG_per_raw<-ggplot(data = CellInfo) + theme_bw() +
  geom_boxplot(aes(x="1",(noCap.unique_CGs/noCap.raw_reads)),fill=noCap) +
  geom_boxplot(aes(x="2",(stdBlock.unique_CGs/stdBlock.raw_reads)),fill=stdBlock) +
  geom_boxplot(aes(x="3",(custBlock.unique_CGs/custBlock.raw_reads)),fill=custBlock) +
  geom_boxplot(aes(x="4",(wash67.unique_CGs/wash67.raw_reads)),fill=wash67) +
  xlab("Condition") + ylab("Unique CGs Covered per Raw Read per Cell")
CG_per_raw
```

```
ggsave(useDingbats=FALSE,filename = "CG_per_raw.pdf",plot=CG_per_raw,width=4,height=4)

global_mCH<-ggplot(data = CellInfo) + theme_bw() +
  geom_boxplot(aes(x="1",noCap.mCH_pct),fill=noCap) +
  geom_boxplot(aes(x="2",stdBlock.mCH_pct),fill=stdBlock) +
  geom_boxplot(aes(x="3",custBlock.mCH_pct),fill=custBlock) +
  geom_boxplot(aes(x="4",wash67.mCH_pct),fill=wash67) +
  xlab("Condition") + ylab("Percent Methylated CHs per Cell") +
  ylim(c(0,1))
global_mCH
```

```
## Warning: Removed 69 rows containing non-finite values (`stat_boxplot()`).
```

```
## Warning: Removed 68 rows containing non-finite values (`stat_boxplot()`).
## Removed 68 rows containing non-finite values (`stat_boxplot()`).
```

```
## Warning: Removed 67 rows containing non-finite values (`stat_boxplot()`).
```

```
ggsave(useDingbats=FALSE,filename = "global_mCH.pdf",plot=global_mCH,width=4,height=4)
```

```
## Warning: Removed 69 rows containing non-finite values (`stat_boxplot()`).
```

```
## Warning: Removed 68 rows containing non-finite values (`stat_boxplot()`).
## Removed 68 rows containing non-finite values (`stat_boxplot()`).
```

```
## Warning: Removed 67 rows containing non-finite values (`stat_boxplot()`).
```

```
global_mCG<-ggplot(data = CellInfo) + theme_bw() +
  geom_boxplot(aes(x="1",noCap.mCG_pct),fill=noCap) +
  geom_boxplot(aes(x="2",stdBlock.mCG_pct),fill=stdBlock) +
  geom_boxplot(aes(x="3",custBlock.mCG_pct),fill=custBlock) +
  geom_boxplot(aes(x="4",wash67.mCG_pct),fill=wash67) +
  xlab("Condition") + ylab("Percent Methylated CGs per Cell")
global_mCG
```

```
ggsave(useDingbats=FALSE,filename = "global_mCG.pdf",plot=global_mCG,width=4,height=4)

target_mCG<-ggplot(data = CellInfo) + theme_bw() +
  geom_boxplot(aes(x="1",noCap.target_mCG_pct),fill=noCap) +
  geom_boxplot(aes(x="2",stdBlock.target_mCG_pct),fill=stdBlock) +
  geom_boxplot(aes(x="3",custBlock.target_mCG_pct),fill=custBlock) +
  geom_boxplot(aes(x="4",wash67.target_mCG_pct),fill=wash67) +
  xlab("Condition") + ylab("On Target Percent Methylated CGs per Cell")
target_mCG
```

```
ggsave(useDingbats=FALSE,filename = "target_mCG.pdf",plot=target_mCG,width=4,height=4)
```

stats

```
Summary<-cbind(apply(CellInfo, 2, median),apply(CellInfo, 2, mean),apply(CellInfo, 2, sd),apply(CellInfo, 2, min),apply(CellInfo, 2, max))
colnames(Summary)<-c("Median","Mean","StDev","Min","Max")
Summary
```

```
##                                   Median         Mean        StDev        Min
## noCap.raw_reads               151845.000 1.761218e+05 1.173813e+05  32179.000
## noCap.trim_reads              138153.000 1.589874e+05 1.034841e+05  31091.000
## noCap.aligned_reads           227115.000 2.603686e+05 1.660292e+05  53817.000
## noCap.unique_reads            213723.000 2.443300e+05 1.550021e+05  50342.000
## noCap.aligned_onTarget         11567.000 1.322980e+04 8.963682e+03   1544.000
## noCap.unique_onTarget          10875.000 1.243453e+04 8.392149e+03   1406.000
## noCap.aligned_enrichment           1.272 1.236887e+00 1.881291e-01      0.619
## noCap.unique_enrichment            1.274 1.238277e+00 1.872458e-01      0.628
## noCap.unique_CGs               93676.000 1.050007e+05 6.367726e+04  19337.000
## noCap.unique_CHs             2148527.000 2.465806e+06 1.457685e+06 485175.000
## noCap.mCG_pct                     79.920 7.833780e+01 5.376503e+00     53.000
## noCap.mCH_pct                      0.540 1.075786e+00 3.787276e+00      0.320
## noCap.unique_CG_onTarget       13431.000 1.512607e+04 9.852230e+03   1384.000
## noCap.target_CG_covPct             0.290 3.304595e-01 2.151901e-01      0.030
## noCap.target_mCG_pct              50.550 5.086626e+01 4.258719e+00     37.450
## custBlock.raw_reads           152119.000 1.770285e+05 1.184565e+05  18449.000
## custBlock.trim_reads          137208.000 1.585998e+05 1.037929e+05  16115.000
## custBlock.aligned_reads       227389.000 2.601230e+05 1.671843e+05  25620.000
## custBlock.unique_reads        175004.000 2.004709e+05 1.265568e+05  19819.000
## custBlock.aligned_onTarget     86459.000 9.813565e+04 6.561220e+04   5482.000
## custBlock.unique_onTarget      55579.000 6.157081e+04 3.880184e+04   3934.000
## custBlock.aligned_enrichment       9.409 9.395876e+00 1.754539e+00      3.628
## custBlock.unique_enrichment        7.676 7.739686e+00 1.556607e+00      2.824
## custBlock.unique_CGs          139006.000 1.528756e+05 9.266301e+04  11526.000
## custBlock.unique_CHs         1849949.000 2.111732e+06 1.251904e+06 164612.000
## custBlock.mCG_pct                 60.230 6.044980e+01 5.114984e+00     43.490
## custBlock.mCH_pct                  0.510 1.000642e+00 3.546038e+00      0.320
## custBlock.unique_CG_onTarget   88572.000 9.659821e+04 6.067777e+04   6424.000
## custBlock.target_CG_covPct         1.940 2.110574e+00 1.325814e+00      0.140
## custBlock.target_mCG_pct          47.560 4.779299e+01 4.016878e+00     34.640
## stdBlock.raw_reads            152981.000 1.770374e+05 1.163417e+05  19601.000
## stdBlock.trim_reads           137802.000 1.581996e+05 1.018860e+05  17473.000
## stdBlock.aligned_reads        226740.000 2.614531e+05 1.655118e+05  27935.000
## stdBlock.unique_reads         175255.000 2.021912e+05 1.259029e+05  21699.000
## stdBlock.aligned_onTarget      84032.000 9.475684e+04 6.247813e+04   5629.000
## stdBlock.unique_onTarget       54453.000 5.972815e+04 3.726427e+04   4033.000
## stdBlock.aligned_enrichment        9.033 9.028870e+00 1.718388e+00      3.394
## stdBlock.unique_enrichment         7.387 7.439763e+00 1.526095e+00      2.647
## stdBlock.unique_CGs           137904.000 1.511471e+05 9.073207e+04  12087.000
## stdBlock.unique_CHs          1865086.000 2.121035e+06 1.247530e+06 181114.000
## stdBlock.mCG_pct                  61.250 6.147067e+01 5.186453e+00     44.190
## stdBlock.mCH_pct                   0.500 1.000721e+00 3.595246e+00      0.300
## stdBlock.unique_CG_onTarget    84916.000 9.319158e+04 5.816237e+04   6408.000
## stdBlock.target_CG_covPct          1.860 2.036298e+00 1.270802e+00      0.140
## stdBlock.target_mCG_pct           48.100 4.839778e+01 4.027840e+00     35.290
## wash67.raw_reads              143931.000 1.783618e+05 1.348448e+05  12392.000
## wash67.trim_reads             127976.000 1.572200e+05 1.172756e+05  11511.000
## wash67.aligned_reads          215226.000 2.633088e+05 1.934405e+05  19480.000
## wash67.unique_reads           154192.000 1.874651e+05 1.342816e+05  16154.000
## wash67.aligned_onTarget        87320.000 1.048409e+05 7.822967e+04   2988.000
## wash67.unique_onTarget         46072.000 5.158261e+04 3.390566e+04   1960.000
## wash67.aligned_enrichment         10.058 9.959909e+00 1.990925e+00      3.385
## wash67.unique_enrichment           7.136 7.148713e+00 1.647075e+00      2.177
## wash67.unique_CGs             134432.000 1.512422e+05 9.761725e+04   8735.000
## wash67.unique_CHs            1576526.000 1.871494e+06 1.257195e+06 124930.000
## wash67.mCG_pct                    57.180 5.755377e+01 5.530147e+00     41.380
## wash67.mCH_pct                     0.490 9.920992e-01 3.583032e+00      0.290
## wash67.unique_CG_onTarget      92725.000 1.009818e+05 6.521977e+04   4110.000
## wash67.target_CG_covPct            2.030 2.206454e+00 1.425041e+00      0.090
## wash67.target_mCG_pct             45.290 4.559606e+01 4.143473e+00     31.580
##                                       Max
## noCap.raw_reads                990205.000
## noCap.trim_reads               886125.000
## noCap.aligned_reads           1420960.000
## noCap.unique_reads            1321499.000
## noCap.aligned_onTarget          75874.000
## noCap.unique_onTarget           70656.000
## noCap.aligned_enrichment            1.696
## noCap.unique_enrichment             1.693
## noCap.unique_CGs               543890.000
## noCap.unique_CHs             12337941.000
## noCap.mCG_pct                      93.940
## noCap.mCH_pct                      61.510
## noCap.unique_CG_onTarget        84080.000
## noCap.target_CG_covPct              1.840
## noCap.target_mCG_pct               86.140
## custBlock.raw_reads            986086.000
## custBlock.trim_reads           854952.000
## custBlock.aligned_reads       1371104.000
## custBlock.unique_reads         988078.000
## custBlock.aligned_onTarget     585986.000
## custBlock.unique_onTarget      328018.000
## custBlock.aligned_enrichment       16.867
## custBlock.unique_enrichment        15.175
## custBlock.unique_CGs           744405.000
## custBlock.unique_CHs         10021841.000
## custBlock.mCG_pct                  90.050
## custBlock.mCH_pct                  60.480
## custBlock.unique_CG_onTarget   500699.000
## custBlock.target_CG_covPct         10.940
## custBlock.target_mCG_pct           84.440
## stdBlock.raw_reads             965681.000
## stdBlock.trim_reads            835047.000
## stdBlock.aligned_reads        1349530.000
## stdBlock.unique_reads          979965.000
## stdBlock.aligned_onTarget      558678.000
## stdBlock.unique_onTarget       317034.000
## stdBlock.aligned_enrichment        13.911
## stdBlock.unique_enrichment         12.682
## stdBlock.unique_CGs            728409.000
## stdBlock.unique_CHs          10091752.000
## stdBlock.mCG_pct                   90.190
## stdBlock.mCH_pct                   60.600
## stdBlock.unique_CG_onTarget    482636.000
## stdBlock.target_CG_covPct          10.550
## stdBlock.target_mCG_pct            83.870
## wash67.raw_reads              1119501.000
## wash67.trim_reads              957379.000
## wash67.aligned_reads          1568932.000
## wash67.unique_reads           1040547.000
## wash67.aligned_onTarget        694492.000
## wash67.unique_onTarget         272006.000
## wash67.aligned_enrichment          15.581
## wash67.unique_enrichment           13.428
## wash67.unique_CGs              758606.000
## wash67.unique_CHs            10808331.000
## wash67.mCG_pct                     89.860
## wash67.mCH_pct                     60.960
## wash67.unique_CG_onTarget      513053.000
## wash67.target_CG_covPct            11.210
## wash67.target_mCG_pct              84.000
```

```
write.table(Summary, quote = FALSE, sep = ",", file = "CellInfo_Summary.csv")
```

Brain data

move to dir, load dependencies & load data

```
setwd("E:\\Data/sciMET_CAP/")
library(ggplot2)
BrainCellInfo<-read.table("BrainCellInfo.txt", header = TRUE)
rownames(BrainCellInfo) <- BrainCellInfo$cellID
BrainCellInfo<-BrainCellInfo[,-1]
```

plots

```
brain_totalCG_by_raw<-ggplot(data = BrainCellInfo) + theme_bw() +
  geom_point(aes(log10(noCap.raw_reads),log10(noCap.unique_CGs)),color=noCap,size=1) +
  geom_point(aes(log10(cap.raw_reads),log10(cap.unique_CGs)),color=cap,size=1) +
  xlab("Log10 Raw Reads per Cell") + ylab(" Log10 Unique CGs per Cell")
brain_totalCG_by_raw
```

```
ggsave(useDingbats=FALSE,filename = "brain_totalCG_by_raw.pdf",plot=brain_totalCG_by_raw,width=4,height=4)

brain_onCG_by_raw<-ggplot(data = BrainCellInfo) + theme_bw() +
  geom_point(aes(log10(noCap.raw_reads),log10(noCap.unique_CG_onTarget)),color=noCap,size=1) +
  geom_point(aes(log10(cap.raw_reads),log10(cap.unique_CG_onTarget)),color=cap,size=1) +
  xlab("Log10 Raw Reads per Cell") + ylab("Log10 On Target CGs per Cell")
brain_onCG_by_raw
```

```
ggsave(useDingbats=FALSE,filename = "brain_onCG_by_raw.pdf",plot=brain_onCG_by_raw,width=4,height=4)

brain_log10_raw<-ggplot(data = BrainCellInfo) + theme_bw() +
  geom_boxplot(aes(x="1",log10(noCap.raw_reads)),fill=noCap) +
  geom_boxplot(aes(x="3",log10(cap.raw_reads)),fill=cap) +
  xlab("Condition") + ylab("log10 Raw Reads per Cell")
brain_log10_raw
```

```
ggsave(useDingbats=FALSE,filename = "brain_log10_raw.pdf",plot=brain_log10_raw,width=4,height=4)

brain_total_CGs<-ggplot(data = BrainCellInfo) + theme_bw() +
  geom_boxplot(aes(x="1",log10(noCap.unique_CGs)),fill=noCap) +
  geom_boxplot(aes(x="3",log10(cap.unique_CGs)),fill=cap) +
  xlab("Condition") + ylab("Log10 CGs Covered per Cell")
brain_total_CGs
```

```
ggsave(useDingbats=FALSE,filename = "brain_total_CGs.pdf",plot=brain_total_CGs,width=4,height=4)

brain_fold_CG_over_noCap<-ggplot(data = BrainCellInfo) + theme_bw() +
  geom_boxplot(aes(x="3",(cap.unique_CGs/noCap.unique_CGs)),fill=cap) +
  xlab("Condition") + ylab("CGs Covered over No Capture per Cell")
brain_fold_CG_over_noCap
```

```
ggsave(useDingbats=FALSE,filename = "brain_fold_CG_over_noCap.pdf",plot=brain_fold_CG_over_noCap,width=4,height=4)

brain_pct_CG_covered<-ggplot(data = BrainCellInfo) + theme_bw() +
  geom_boxplot(aes(x="1",noCap.target_CG_covPct),fill=noCap) +
  geom_boxplot(aes(x="3",cap.target_CG_covPct),fill=cap) +
  xlab("Condition") + ylab("Percent Target CGs Covered per Cell") +
  ylim(c(0,5))
brain_pct_CG_covered
```

```
## Warning: Removed 2 rows containing non-finite values (`stat_boxplot()`).
```

```
## Warning: Removed 356 rows containing non-finite values (`stat_boxplot()`).
```

```
ggsave(useDingbats=FALSE,filename = "brain_pct_CG_covered.pdf",plot=brain_pct_CG_covered,width=4,height=4)
```

```
## Warning: Removed 2 rows containing non-finite values (`stat_boxplot()`).
## Removed 356 rows containing non-finite values (`stat_boxplot()`).
```

```
brain_aligned_enrichment<-ggplot(data = BrainCellInfo) + theme_bw() +
  geom_boxplot(aes(x="1",noCap.aligned_enrichment),fill=noCap) +
  geom_boxplot(aes(x="3",cap.aligned_enrichment),fill=cap) +
  xlab("Condition") + ylab("Aligned Read Target Enrichment per Cell")
brain_aligned_enrichment
```

```
ggsave(useDingbats=FALSE,filename = "brain_aligned_enrichment.pdf",plot=brain_aligned_enrichment,width=4,height=4)

brain_unique_enrichment<-ggplot(data = BrainCellInfo) + theme_bw() +
  geom_boxplot(aes(x="1",noCap.unique_enrichment),fill=noCap) +
  geom_boxplot(aes(x="3",cap.unique_enrichment),fill=cap) +
  xlab("Condition") + ylab("Unique Read Target Enrichment per Cell")
brain_unique_enrichment
```

```
ggsave(useDingbats=FALSE,filename = "brain_unique_enrichment.pdf",plot=brain_unique_enrichment,width=4,height=4)

brain_pct_unique_reads<-ggplot(data = BrainCellInfo) + theme_bw() +
  geom_boxplot(aes(x="1",((noCap.unique_reads/noCap.aligned_reads)*100)),fill=noCap) +
  geom_boxplot(aes(x="3",((cap.unique_reads/cap.aligned_reads)*100)),fill=cap) +
  xlab("Condition") + ylab("Percent Unique Reads of Total Aligned per Cell")
brain_pct_unique_reads
```

```
ggsave(useDingbats=FALSE,filename = "brain_pct_unique_reads.pdf",plot=brain_pct_unique_reads,width=4,height=4)

brain_CG_per_raw<-ggplot(data = BrainCellInfo) + theme_bw() +
  geom_boxplot(aes(x="1",(noCap.unique_CGs/noCap.raw_reads)),fill=noCap) +
  geom_boxplot(aes(x="3",(cap.unique_CGs/cap.raw_reads)),fill=cap) +
  xlab("Condition") + ylab("Unique CGs Covered per Raw Read per Cell")
brain_CG_per_raw
```

```
ggsave(useDingbats=FALSE,filename = "brain_CG_per_raw.pdf",plot=brain_CG_per_raw,width=4,height=4)

brain_global_mCH<-ggplot(data = BrainCellInfo) + theme_bw() +
  geom_violin(aes(x="1",noCap.mCH_pct),fill=noCap) +
  geom_violin(aes(x="3",cap.mCH_pct),fill=cap) +
  xlab("Condition") + ylab("Percent Methylated CHs per Cell") +
  ylim(c(0,20))
brain_global_mCH
```

```
## Warning: Removed 93 rows containing non-finite values (`stat_ydensity()`).
```

```
## Warning: Removed 90 rows containing non-finite values (`stat_ydensity()`).
```

```
ggsave(useDingbats=FALSE,filename = "brain_global_mCH.pdf",plot=brain_global_mCH,width=4,height=4)
```

```
## Warning: Removed 93 rows containing non-finite values (`stat_ydensity()`).
## Removed 90 rows containing non-finite values (`stat_ydensity()`).
```

```
brain_global_mCG<-ggplot(data = BrainCellInfo) + theme_bw() +
  geom_boxplot(aes(x="1",noCap.mCG_pct),fill=noCap) +
  geom_boxplot(aes(x="3",cap.mCG_pct),fill=cap) +
  xlab("Condition") + ylab("Percent Methylated CGs per Cell")
brain_global_mCG
```

```
ggsave(useDingbats=FALSE,filename = "brain_global_mCG.pdf",plot=brain_global_mCG,width=4,height=4)

brain_target_mCG<-ggplot(data = BrainCellInfo) + theme_bw() +
  geom_boxplot(aes(x="1",noCap.target_mCG_pct),fill=noCap) +
  geom_boxplot(aes(x="3",cap.target_mCG_pct),fill=cap) +
  xlab("Condition") + ylab("On Target Percent Methylated CGs per Cell")
brain_target_mCG
```

```
ggsave(useDingbats=FALSE,filename = "brain_target_mCG.pdf",plot=brain_target_mCG,width=4,height=4)
```

brains ummary

```
Brain_Summary<-cbind(apply(BrainCellInfo, 2, median),apply(BrainCellInfo, 2, mean),apply(BrainCellInfo, 2, sd),apply(BrainCellInfo, 2, min),apply(BrainCellInfo, 2, max))
colnames(Brain_Summary)<-c("Median","Mean","StDev","Min","Max")
Brain_Summary
```

```
##                                Median         Mean        StDev        Min
## noCap.raw_reads           227591.5000 3.230259e+05 2.778034e+05  15046.000
## noCap.trim_reads          210371.5000 2.923382e+05 2.434002e+05  12346.000
## noCap.aligned_reads       339674.0000 4.529717e+05 3.593736e+05  18801.000
## noCap.unique_reads        320944.5000 4.266647e+05 3.372613e+05  17892.000
## noCap.aligned_onTarget     23155.5000 3.126032e+04 2.642410e+04    624.000
## noCap.unique_onTarget      21862.0000 2.941574e+04 2.478646e+04    603.000
## noCap.aligned_enrichment       1.7260 1.666416e+00 2.787949e-01      0.757
## noCap.unique_enrichment        1.7220 1.664752e+00 2.773814e-01      0.777
## noCap.unique_CGs          172785.0000 2.197236e+05 1.695270e+05   5951.000
## noCap.unique_CHs         3618968.5000 4.469984e+06 3.272886e+06 156297.000
## noCap.mCG_pct                 78.0800 7.889051e+01 5.615831e+00     59.190
## noCap.mCH_pct                  1.9200 8.595857e+00 1.870717e+01      0.710
## noCap.unique_CG_onTarget   32204.0000 4.155838e+04 3.384067e+04    620.000
## noCap.target_CG_covPct         0.7050 9.079714e-01 7.393712e-01      0.010
## noCap.target_mCG_pct          45.5200 4.786335e+01 1.183161e+01     23.220
## cap.raw_reads             238261.0000 3.230259e+05 2.855014e+05   3877.000
## cap.trim_reads            226771.0000 3.011054e+05 2.622411e+05   3646.000
## cap.aligned_reads         384416.0000 5.007268e+05 4.257093e+05   6217.000
## cap.unique_reads          336692.5000 4.367255e+05 3.676628e+05   5953.000
## cap.aligned_onTarget      111733.5000 1.502254e+05 1.340129e+05    645.000
## cap.unique_onTarget        84704.0000 1.123945e+05 9.698230e+04    600.000
## cap.aligned_enrichment         7.6705 7.267630e+00 1.960042e+00      1.205
## cap.unique_enrichment          6.6260 6.326366e+00 1.672027e+00      1.168
## cap.unique_CGs            269011.0000 3.481738e+05 2.887092e+05   3170.000
## cap.unique_CHs           3797181.0000 4.693704e+06 3.754253e+06  59281.000
## cap.mCG_pct                   63.4100 6.478557e+01 8.452307e+00     39.790
## cap.mCH_pct                    1.8400 7.869657e+00 1.724411e+01      0.720
## cap.unique_CG_onTarget    154870.0000 2.007112e+05 1.698398e+05    798.000
## cap.target_CG_covPct           3.3850 4.385667e+00 3.710956e+00      0.020
## cap.target_mCG_pct            48.0400 4.923616e+01 8.861448e+00     26.350
##                                   Max
## noCap.raw_reads           2012197.000
## noCap.trim_reads          1739632.000
## noCap.aligned_reads       2742692.000
## noCap.unique_reads        2578279.000
## noCap.aligned_onTarget     197597.000
## noCap.unique_onTarget      185295.000
## noCap.aligned_enrichment        2.866
## noCap.unique_enrichment         2.850
## noCap.unique_CGs          1326036.000
## noCap.unique_CHs         26728395.000
## noCap.mCG_pct                  97.350
## noCap.mCH_pct                  93.860
## noCap.unique_CG_onTarget   256904.000
## noCap.target_CG_covPct          5.610
## noCap.target_mCG_pct           94.940
## cap.raw_reads             2270843.000
## cap.trim_reads            2129662.000
## cap.aligned_reads         3582444.000
## cap.unique_reads          3033935.000
## cap.aligned_onTarget      1193804.000
## cap.unique_onTarget        841500.000
## cap.aligned_enrichment         13.728
## cap.unique_enrichment          12.272
## cap.unique_CGs            2370744.000
## cap.unique_CHs           31940069.000
## cap.mCG_pct                    95.850
## cap.mCH_pct                    92.180
## cap.unique_CG_onTarget    1367457.000
## cap.target_CG_covPct           29.880
## cap.target_mCG_pct             95.070
```

```
write.table(Brain_Summary, quote = FALSE, sep = ",", file = "BrainCellInfo_Summary.csv")
```
