# Supplementary material for: sciMET-cap: high-throughput single-cell methylation analysis with a reduced sequencing burden
Source: Genome Biol. 2024 Jul 10;25:186. doi: 10.1186/s13059-024-03306-7 (PMC11234687; doi:10.1186/s13059-024-03306-7)
Supplement: Supplementary file 5 — Additional file 5. Review history. [file 13059_2024_3306_MOESM5_ESM.docx]

Review History

**First round of review**

**Reviewer 1**

**Were you able to assess all statistics in the manuscript, including the appropriateness of statistical tests used?**

**Yes:** acceptable

**Were you able to directly test the methods?**

No

**Comments to author:**

Genome-wide shotgun profiling of DNA methylation in single cells requires a large amount of sequencing (~2 million reads per cell), which drives up the cost. The manuscript addresses a significant need by integrating the recently published sci-METv2 method and off-the-shelf post-bisulfite methylation capture reagent from Twist Bioscience. The performance of the method seems moderate, with a 7-8 fold enrichment of a 123Mb capture region, which translates to a 25% to 30% on-target rate. The on-target rate is much lower than capture on a 4-base non-bisulfite-converted genome (e.g. exome sequencing), which typically yields >80% on-target rate. However, capture on a 3-base bisulfite-converted genome may be intrinsically inefficient and less specific. There are also few options for post-bisulfite capture reagents, if Twist Bioscience is not the only one, it is difficult to perform a comparison of different vendors. So although the reported technology is moderately successful, the method perhaps represents the best possible solution and is therefore suitable for publication in GB with some improvements to the manuscript.

1.The manuscript should be more thorough in discussing the metrics of the methylation capture and comparing the performance of the Twist capture probes to NimbleGen post-bisulfite capture probes described in Li et al. 2015. NAR (PMID: 25813045). Sequencing metadata was provided in "AdditonalFile2" but was not adequately discussed in the figures and the main text.

2. The manuscript should more explicitly discuss the on-target rate instead of focusing on fold-enrichment. The discussion section also briefly mentioned "approximately half of all sequence reads falling within the 125 Mb target capture region". How was the ~50% number calculated? A quick calculation based on 125 Mb / 3G genome size * (7 to 8 folds enrichment) = ~25% to 30% instead of 50%.

3. The manuscript did not adequately address the concern that capturing might preferentially recover methylated or unmethylated cytosines. The authors should compare at a single base resolution, and in various GC content regions, the methylation level of cytosines in the capture region with or without capture. Coverage uniformity on both strands should also be analyzed.

4. sciMET-cap data contains a large amount of data from genomic regions not targeted for capture. A more thorough comparison is needed to ensure the methylation data from non-targeted regions are equivalent to sciMETv2 without capture.

5. The manuscript should address whether sciMET-cap now enables direct comparisons between single cells for cytosines (within the targeted region) with coverage in multiple cells. Direct comparison of cytosines between single cells has been challenging due to the sparsity of single-cell methylome leading to an extraordinarily low probability that a cytosine is covered in more than 1 cell. But perhaps the frequency increases significantly with capture? This feature is particularly relevant in applications such as DNA methylation clock.

**Reviewer 2**

**Were you able to assess all statistics in the manuscript, including the appropriateness of statistical tests used?**

**Yes:** The authors have used appropriate and established statistical tests

**Were you able to directly test the methods?**

No

**Comments to author:**

The first methods for genome-wide profiling of DNA methylation at single-cell resolution were reported nearly ten years ago. Despite a number of advances in methods to enable increased through-put since then, single-cell DNA methylation analysis for truly high-throughput studies has been difficult and expensive to implement. Thus, it lags behind scRNA-seq or scATAC-seq, which have become far more routine applications, enabling for example, the construction of high-resolution cell atlas that incorporate millions of cells, and which have been applied in numerous studies of cell architecture and cell heterogeneity both in normal development and disease processes. This study seeks to combine two innovations to improve single-cell bisulphite sequencing output in terms of throughput and costs. 1) Combinatorial cell barcoding that allows large numbers of individually-labelled nuclei to be processed in bulk, thus avoiding some of the technical anomalies seen in processing individual cells, as well as handling: this has been 'solved' in previous work from this group (Mulqueen et al. 2018; Nichols et al. 2022).
2) Target capture, to enable prioritisation of genome features of particular interest (regulatory features) and reduce wastage of sequencing effort of uninformative genomic stretches. In essence, the study adds an off-the-shelf target capture component to the sciMETv2 workflow recently described in Nichols et al. 2022. Reducing the genomic space interrogated should also increase the possibility of concordant information sampling between cells, assuming sufficiently complex representation at the initial library generation at the individual cell level.

This is a technical paper that explores some of the parameters in the target capture - which employs the human methylome panel form Twist Biosciences - and the impact on library output. The authors generate data from 2000-6000 cell PBMCs or human frontal gyrus cells. They are able to reference against a previous sciMET library from the same human brain sample. Performance of the capture libraries against non-captured libraries is evaluated in relation to cell clustering and numbers of differentially methylated regions (DMRs) called, with the premise that clarity of clustering is the major success outcome. On this basis, obtaining comparable clustering from the combined 350 million sequence reads from a capture library compared with 7 billion from a non-captured library is an impressive advance.

I have few specific comments.

The sciMET-cap libraries are reported as yielding 154,000-175,000 unique reads per cell, covering 134,000-139,000 unique CpG sites. For completeness of information, it would be useful to give this also as a % of CpG sites on the capture (which is 3.98 million? so ~3.5%).

The authors comment that only about half of all reads map to the target capture regions. They regard this as a benefit in that the off-target reads can be used to generate whole-genome methylomes for cell types after bulking the data. Is this the expectation from the capture arrays or a function of a reduced input of DNA applied? The point is that if the off-target capture was reduced to <10%, there would be the potential for an ~2-fold reduction in sequencing cost per study, or for greater coverage of capture sites per cell perhaps. An explanation for the 50% off-target rate would be useful for readers.

Capture has the potential limitation of availability of capture libraries. Human methylome studies are clearly well served by off-the-shelf reagents, but researchers interested in other species will require custom reagents, which might be a barrier to some.

**Reviewer 3**

**Were you able to assess all statistics in the manuscript, including the appropriateness of statistical tests used?**

Yes

**Were you able to directly test the methods?**

Yes

**Comments to author:**

The authors present sciMET-cap, an improvement over their previous single-cell DNA methylation (DNAm) protocol where they use target enrichment to capture more information per cell with less sequencing reads. This is an important work, as single-cell DNAm assays have not evolved as fast as compared to other omics due to the lack of a robust commercial technology and the associated sequencing. sciMET-cap significantly reduces the sequencing costs and makes DNAm profiling more accessible to research groups. The manuscript is clearly written and the benchmarking is thorough. Also, I appreciate that the authors have made the data available for the reviewers. I only have a few minor comments that I believe could improve the manuscript:

- At lines 6-7 and 242-247 the authors state that per-cluster full-coverage methylomes can be generated from the off-target reads. This is a very useful feature of the method but I cannot find this result in the main text or figures.

- At lines 177-179 the authors state "DMR calling produced a total of 195,483 called regions (Fig. 3c) which were achieved using a total raw read count of 1.7 billion - a 1.5-fold increase in raw sequencing when compared to the input PBMC library sequenced without capture that was able to produce only 5,914 DMRs using the same significance criteria, for a 33-fold improvement." Clearly this is an improvement but it would be useful to make a fairer comparison by downsampling the captured data to the same depth.

- At line 215 the authors state "Finally, we prepared a sciMET-cap library from a separate individual using a single sciMETv2 preparation". Could the authors specify which tissue was used, and which version of sciMETv2: SL or LA?

- I like having a Figure that summarises the approach (Figure 6), but I find it hard to follow with all the UMAPs, arrows and colour schemes. Could the authors simplify the figure and make it more clear to the reader?

- A significant part of the results involve computational work. The authors should make the code accessible for everyone to reproduce the main results shown in the manuscript.

**Authors Response**

**Point-by-point responses to the reviewers’ comments:**

**Response to Reviewers**

Reviewer comments in black, responses in blue. Changes to the manuscript text are quoted in *“blue italic”*.

We thank the three reviewers for their constructive comments. We have incorporated each of the suggested analyses and believe that the manuscript has improved as a result of the feedback.

**Reviewer #1:** Genome-wide shotgun profiling of DNA methylation in single cells requires a large amount of sequencing (~2 million reads per cell), which drives up the cost. The manuscript addresses a significant need by integrating the recently published sci-METv2 method and off-the-shelf post-bisulfite methylation capture reagent from Twist Bioscience. The performance of the method seems moderate, with a 7-8 fold enrichment of a 123Mb capture region, which translates to a 25% to 30% on-target rate. The on-target rate is much lower than capture on a 4-base non-bisulfite-converted genome (e.g. exome sequencing), which typically yields >80% on-target rate. However, capture on a 3-base bisulfite-converted genome may be intrinsically inefficient and less specific. There are also few options for post-bisulfite capture reagents, if Twist Bioscience is not the only one, it is difficult to perform a comparison of different vendors. So although the reported technology is moderately successful, the method perhaps represents the best possible solution and is therefore suitable for publication in GB with some improvements to the manuscript.

We thank the reviewer for the positive comments. We believe that the additional analyses and framing of the results suggested by the reviewer have led to an improved manuscript. We first want to address the overall goals of this study. While achieving a very high on-target rate / fold enrichment could be beneficial for a number of applications, our primary goal is to reduce the sequencing burden for studies that aim to produce high cell counts with relatively low coverage per-cell. We explicitly state this as the goal in the title of the manuscript “...*with a reduced sequencing burden*”, but agree that we could be more explicit in the introduction as well as elaborate further on this point in the discussion.

Background: *“We reasoned that the enrichment of putative regulatory regions that likely exhibit the greatest cell type variability may lower the minimum number of sequence reads required to achieve cell type identification and allow for a greater number of cells to be profiled at an equivalent cost point.”*

Discussion: *“Captured libraries resulted in a median of 28-31% of all sequence reads falling within the 125 Mbp target capture region (7-8-fold enrichment), reaching 43% on target when including regions within 200 bp of target probe sites (referred to as “shadow coverage”), leaving the remaining reads distributed throughout the genome. This enables the use of the enriched on-target regions for cell type assignment followed by aggregation of cells within a cluster to produce near full-coverage methylomes. We then evaluated the ability of each condition to capture differentially methylated loci, which produced consistent results across capture conditions. Consistent results for strand uniformity and methylation levels were also observed between capture and non-capture libraries, confirming that the capture process itself does not skew methylation calls.*

*Notably, the capture rate falls below what is typical for bulk libraries, which achieve approximately twice the enrichment, at 12-18-fold. Our attempts to optimize by leveraging custom blockers and a more stringent wash temperature did not achieve meaningful improvements, suggesting alternative approaches may be required. This may involve modifications to the sciMETv2 workflow to produce library fragments with a larger insert size which would be more favorable to hybridization, or possibly a re-design of the capture probes to increase their binding affinity. Such improvements may yield a greater on target rate and enable applications where high coverage within the target region can be obtained for individual cells at a reasonable cost point. However, sciMET-cap as it stands greatly reduces sequencing costs for studies that seek to profile a large number of cells efficiently.”*

To summarize, we agree that greater fold enrichment would be ideal; as there is always the option of mixing pre- and post-capture libraries to achieve any balance of on vs off target reads. However, we have tested all of the available parameters for increasing on-target rates and were not able to improve beyond 7-8-fold / ~25­30% on target. As the reviewer states, we believe that this “represents the best possible solution” and regard an ~8-10-fold reduction in sequencing cost per cell for high-cell-count studies as a valuable result.

We next want to address the calculations of fold enrichment and on-target rates. The reviewer brings up important points on this topic, and we completely agree that additional clarification is needed, as well as additional context regarding post-bisulfite capture.

First, it is true that 3-base capture is less efficient than non-converted 4-base capture. To summarize bulk capture results, Twist reports an enrichment range of 12-18-fold and note that this can result in an on-target rate of 95%. However, these values include “shadow coverage” in their calculation, which includes 200 bp up and down stream of each probe site. Many target sites are short (median 120 bp; 75% of target sites ≤ 220 bp), therefore this expansion results in a final, merged target size of >310 Mbp. If a hard cutoff is used for an on-target rate (excluding “shadow coverage”, hereafter referred to as ‘hard target’), this results in a range of 50-74% for the bulk assay.

In our initial report of our on-target rate (-50%), we also included “shadow coverage” and was calculated pre duplicate removal, as it was generated using the Twist computational workflow; however, our fold enrichment was calculated using the hard target. Much of this discrepancy is due to ways in which companies find metrics that make their assays come across in the most favorable light. With the goal of transparency, we believe that the ‘hard target’ definition is more appropriate and representative, which indeed makes our on-target rate fall between 25-30%. Specifically, we now report the median on-target percentage for each capture condition which ranges from 28.4-30.6% and is reflected in the results (below, point 2) and discussion sections (above).

1.The manuscript should be more thorough in discussing the metrics of the methylation capture and comparing the performance of the Twist capture probes to NimbleGen post-bisulfite capture probes described in Li et al. 2015. NAR (PMID: 25813045).

Additional options for post-bisulfite capture would be ideal; however, the referenced NimbleGen (Roche) kit for post-bisulfite capture has been discontinued and is therefore no longer available making a direct performance comparison impossible. (At the onset of this project we did attempt a capture using a single set of Roche probes we had left over from a prior study that were -4 years expired but it failed to produce a library.) We did explore other vendors, including Arbor Bioscience; however, the generation of a probe set similar to the Twist panel would cost at a minimum >$100,000 USD, making it prohibitively expensive for this study (quoted pricing below):

| Arbor Bioscience | 16Rxn | 48Rxn | 96Rxn | 384Rxn |
| --- | --- | --- | --- | --- |
| myBaits Methyl-Seq 1020-1040k | $110,448 | $135,980 | $184,704 | $536,900 |

Sequencing metadata was provided in "AdditonalFile2" but was not adequately discussed in the figures and the main text.

In the current main text, we note a number of specific metrics pulled from these files, citing median values, but agree that an explicit statement in the main text that points to the detailed summaries and per-cell statistics is warranted. We now include the following callout at the end of the “Evaluation of sciMET-cap target enrichment conditions” results section:

Results: *“Summary metrics and per-cell values for these assessments can be found in Additional Files 1 and 2, respectively.”*

2. The manuscript should more explicitly discuss the on-target rate instead of focusing on fold-enrichment. The discussion section also briefly mentioned "approximately half of all sequence reads falling within the 125 Mb target capture region". How was the -50% number calculated? A quick calculation based on 125 Mb / 3G genome size * (7 to 8 folds enrichment) = -25% to 30% instead of 50%.

We again thank the reviewer for pointing out this discrepancy and refer to the comments above where we now leverage only the hard on-target numbers as opposed to the inclusion of “shadow coverage”.

Results: *“However, the condition with a wash at 67°C exhibited a greater target enrichment of aligned reads with a median of 10.1-fold (40.1% median on target) versus 9.3 (36.0%) and 9.4 (37.5%) for stdBlock and custBlock, respectively. This improvement was eliminated when assessing only unique reads, with enrichment values dropping to a median of 7.4 (29.4%), 7.7 (30.6%) and 7.1-fold (28.4%) for stdBlock, custBlock and wash67, respectively; ...”*

*“This resulted in 4,037 single cell methylomes with a median of 233,400 raw reads per cell, producing a median of 166,270 unique CG sites covered per cell with a median unique read target enrichment of 7.2 (28.7% median on target).”*

*“Similar to the results of the PBMC experiments, the cap condition produced a median aligned read enrichment of 7.7 (30.6% on target) with a median of 1.29 unique CG sites covered per raw sequence read versus 0.75 for the noCap condition (Additional file 1,3).”*

3. The manuscript did not adequately address the concern that capturing might preferentially recover methylated or unmethylated cytosines. The authors should compare at a single base resolution, and in various GC content regions, the methylation level of cytosines in the capture region with or without capture. Coverage uniformity on both strands should also be analyzed.

We thank the reviewer for suggesting the additional analyses. We agree that they are important metrics for the evaluation of possible biases due to the capture. We were pleased to find that for each analysis, the capture did not appear to introduce any bias.

We first assessed strand coverage bias as suggested, which also showed little variation with respect to capture conditions, breaking down evenly across each strand and conversion status. This analysis is also detailed in the main text and included as additional panels in Supplementary Figure 1.

Supplementary Figure 1, New Panels:

Percentage of aligned reads to each strand (Watson or Crick) and genome conversion (C2T or G2A – based on original or copied strand post-bisulfite) is shown for each condition for all reads, reads only present in off-target regions, or reads that overlap the target regions.

We also now include an individual CG-level correlation analysis between samples for all CG sites, on target sites, off target sites, sites in the top 25% of GC-content genomic regions and sites in the bottom 25% of GC-content. This analysis is now detailed in the main text and is accompanied by a new Supplementary Figure 2.

Results: *“Coverage was observed to be uniformly distributed between the Watson and Crick strands as well as the conversion status (C to T or G to A) when comparing conditions, which was true for all reads as well as reads aligning to either on or off target genomic regions, suggesting the capture process does not introduce a strand bias (Fig. S1). Site-level methylation was then evaluated for all CGs, producing Pearson correlations ≥ 0.96 across all conditions, with similar correlations for on target (≥ 0.97) and off target (≥ 0.93) CG sites, as well as for CGs within the top 25% GC-content of 2 kbp bins (≥ 0.97) and bottom 25% (≥ 0.91); confirming that the capture process does not alter base-level methylation calls (Fig. S2).”*

Pearson correlation of the methylation levels of individual CG sites between each condition are shown for all CGs, on target CGs, off target CGs, CGs in regions with high GC content (top 25% of 2 kbp windows) and low GC content (bottom 25% of 2 kbp windows).

4. sciMET-cap data contains a large amount of data from genomic regions not targeted for capture. A more thorough comparison is needed to ensure the methylation data from non-targeted regions are equivalent to sciMETv2 without capture.

We agree with the reviewer and refer to the additional analyses that were performed in the previous response, split by on vs off target ensure that both components are not biased by the capture process itself.

5. The manuscript should address whether sciMET-cap now enables direct comparisons between single cells for cytosines (within the targeted region) with coverage in multiple cells. Direct comparison of cytosines between single cells has been challenging due to the sparsity of single-cell methylome leading to an extraordinarily low probability that a cytosine is covered in more than 1 cell. But perhaps the frequency increases significantly with capture? This feature is particularly relevant in applications such as DNA methylation clock.

We agree that maximizing the number of overlapping CG sites between multiple cells is important for some applications. In any workflow, capture or not, this would require substantial amounts of sequencing per cell and have a library with very high complexity (unique molecules in the pool). Without capture, our sciMETv2.LA approach can achieve relatively high per-cell coverage, with high-performing cells reaching >50% CG coverage with the ability to continue sequencing deeper before hitting excessive diminishing returns (see Nichols et al Nature Communications 2022); however, this requires excessive sequencing. When capture is performed, more sequenced reads are on target, yet the theoretical maximum is still bound by the input library complexity, which is also decreased as a part of the capture. Taken together, sciMETv2.LA with capture and a

high amount of sequencing may produce somewhat improved frequencies of pairwise individual CG site overlap, yet still at a very high sequencing cost.

For a significant improvement at a reasonable cost-point, a greater percent of reads on target would need to be achieved, likely either comparable to, or better than bulk capture. To achieve this gain, it is likely that the target Tm of probes would need to be increased to enable a higher wash temperature without loss in complexity (as was observed with the 67°C wash temperature condition). This would require an entire re-design of the probe set (confirmed by discussions with Twist), which is beyond the scope (and certainly budget) of this manuscript. With the commercial release of sciMETv2, it is possible that adoption of sciMET-cap may motivate Twist to pursue a design optimized for single-cell applications, but until then, we believe we have achieved the best possible outcome.

**Reviewer #2:** The first methods for genome-wide profiling of DNA methylation at single-cell resolution were reported nearly ten years ago. Despite a number of advances in methods to enable increased through-put since then, single-cell DNA methylation analysis for truly high-throughput studies has been difficult and expensive to implement. Thus, it lags behind scRNA-seq or scATAC-seq, which have become far more routine applications, enabling for example, the construction of high-resolution cell atlas that incorporate millions of cells, and which have been applied in numerous studies of cell architecture and cell heterogeneity both in normal development and disease processes. This study seeks to combine two innovations to improve single-cell bisulphite sequencing output in terms of throughput and costs.

1) Combinatorial cell barcoding that allows large numbers of individually-labelled nuclei to be processed in bulk, thus avoiding some of the technical anomalies seen in processing individual cells, as well as handling: this has been 'solved' in previous work from this group (Mulqueen et al. 2018; Nichols et al. 2022).
2) Target capture, to enable prioritization of genome features of particular interest (regulatory features) and reduce wastage of sequencing effort of uninformative genomic stretches. In essence, the study adds an off-the-shelf target capture component to the sciMETv2 workflow recently described in Nichols et al.

2022. Reducing the genomic space interrogated should also increase the possibility of concordant information sampling between cells, assuming sufficiently complex representation at the initial library generation at the individual cell level.

This is a technical paper that explores some of the parameters in the target capture - which employs the human methylome panel form Twist Biosciences - and the impact on library output. The authors generate data from 2000-6000 cell PBMCs or human frontal gyrus cells. They are able to reference against a previous sciMET library from the same human brain sample. Performance of the capture libraries against non-captured libraries is evaluated in relation to cell clustering and numbers of differentially methylated regions (DMRs) called, with the premise that clarity of clustering is the major success outcome. On this basis, obtaining comparable clustering from the combined 350 million sequence reads from a capture library compared with 7 billion from a non-captured library is an impressive advance.

We thank the reviewer for the positive comments and appreciate the valuable suggestions that we believe improve the manuscript.

I have few specific comments.

The sciMET-cap libraries are reported as yielding 154,000-175,000 unique reads per cell, covering 134,000­139,000 unique CpG sites. For completeness of information, it would be useful to give this also as a % of CpG sites on the capture (which is 3.98 million? so ~3.5%).

The covered unique CG sites is across the entire genome versus just on-target, therefore the percent on-target CGs captured is a bit lower with a median just below 2% for each condition. This analysis is shown in Supplementary Figure 1, as well as the mean and median values in Additional File 1 and per-cell values in Additional Files 2 and 3. Notably, these values are for the downsampled read-count-matched datasets, so additional sequencing would yield higher coverage; however, the covered CGs in these data are what we would target in a typical experiment where high cell counts are prioritized over per-cell coverage.

Supplementary Figure 1, Percent on-target CG sites covered panel:

The authors comment that only about half of all reads map to the target capture regions. They regard this as a benefit in that the off-target reads can be used to generate whole-genome methylomes for cell types after bulking the data. Is this the expectation from the capture arrays or a function of a reduced input of DNA applied? The point is that if the off-target capture was reduced to <10%, there would be the potential for an ~2-fold reduction in sequencing cost per study, or for greater coverage of capture sites per cell perhaps. An explanation for the 50% off-target rate would be useful for readers.

Based on suggestions from Reviewer 1, we have revised the calculations for percent of reads on target to only include reads that directly overlap individual probes as opposed to including a buffer on either end of probe targets (which is what Twist uses as their standard for ‘on-target’ which includes “shadow coverage”), this reduces the on-target percent to ~30%. Notably, the probes are designed to capture regions beyond the actual probe sequence, so some of the ‘off-target’ that falls near probes (i.e. “shadow coverage”) is valuable.

Capture has the potential limitation of availability of capture libraries. Human methylome studies are clearly well served by off-the-shelf reagents, but researchers interested in other species will require custom reagents, which might be a barrier to some.

We agree completely. Twist has stated to us that they would produce a mouse methylome panel should there be enough interest, and we hope that this study will help achieve those goals. To that end, we have added the following text:

Discussion: *“Currently, this capture panel is only available for human applications, limiting its application space, though expanding panels to other species, such as mouse, is possible.”*

**Reviewer #3:** The authors present sciMET-cap, an improvement over their previous single-cell DNA methylation (DNAm) protocol where they use target enrichment to capture more information per cell with less sequencing reads. This is an important work, as single-cell DNAm assays have not evolved as fast as compared to other omics due to the lack of a robust commercial technology and the associated sequencing. sciMET-cap significantly reduces the sequencing costs and makes DNAm profiling more accessible to research groups. The manuscript is clearly written and the benchmarking is thorough. Also, I appreciate that the authors have made the data available for the reviewers. I only have a few minor comments that I believe could improve the manuscript:

- At lines 6-7 and 242-247 the authors state that per-cluster full-coverage methylomes can be generated from the off-target reads. This is a very useful feature of the method but I cannot find this result in the main text or figures.

We thank the reviewer for pointing out this oversight. In general, the CG coverage tracks with the number of cells within the cluster, though we note that on an individual CG level, coverage >90% is not reached for any cluster with fewer than 600 cells. However, for purposes of DMR calling, most genomic windows reach a coverage of at least 10 covered CG sites, which is the minimum threshold for DMR calling. We have adjusted our language on this point to and include a new panel in Figure 2 that reflects the coverage achieved for individual CG sites globally and on target, as well as the percent of 1,500 bp windows that pass our DMR calling threshold.

Abstract: *“Accumulated off-target coverage enables genome-wide differentially methylated region (DMR) calling for clusters with as few as 115 cells.”*

Results: *“Aggregated coverage from all cells within each cluster produced a median of 60.1% coverage of individual CG sites across both strands, correlating with cell counts within each cluster. For purposes of DMR calling, we calculated the percentage of 1,500 bp windows with a minimum CG coverage of 10, resulting in a median of 96.0% of windows passing this cutoff, with only one cluster falling below 90% (P8, one of 4 CD4+ T-Cell clusters, 71.6%), suggesting nearly the entire methylome can be assessed for differentially methylated regions using aggregated cell coverage within clusters (Fig. 3c).”*

Figure Legend: *“c. Percent of individual CG sites covered by aggregate cell coverage for each cluster colored by cluster identity in a. (left), for all CG sites on target (middle), and percent of 1,500 bp windows used in DMR calling with a minimum coverage of 10 (right).”*

- At lines 177-179 the authors state "DMR calling produced a total of 195,483 called regions (Fig. 3c) which were achieved using a total raw read count of 1.7 billion - a 1.5-fold increase in raw sequencing when compared to the input PBMC library sequenced without capture that was able to produce only 5,914 DMRs using the same significance criteria, for a 33-fold improvement." Clearly this is an improvement but it would be useful to make a fairer comparison by downsampling the captured data to the same depth.

The reviewer brings up a good point – in the referenced statement we were comparing two datasets with a similar read count (1.7 B vs 1.13 B) yet very different cell counts (~6k capture, ~2k shotgun). If we downsampled the 1.7 billion reads for the ~6k sciMET-cap cells down to the same read count as the ~2k shotgun cells, the coverage per cell would be very different, as would cell counts. Alternatively, we could select only the ~2k cells for which we have both shotgun and sciMET-cap data, but we would be using all of the reads from the ~2k capture cells (501 M) and would then need to downsample the ~2k shotgun cells to the same number which ends up close to the earlier results section where we compare noCap to stdCap (350 M reads for all conditions). Ultimately, we agree that this comparison was not appropriate and have removed it from the manuscript.

- At line 215 the authors state "Finally, we prepared a sciMET-cap library from a separate individual using a single sciMETv2 preparation". Could the authors specify which tissue was used, and which version of sciMETv2: SL or LA?

We appreciate the reviewer pointing out this oversight. We now specify additional details of the preparation in the main text:

Results: *“Finally, we prepared a sciMET-cap library from the same brain region of a separate individual (cortex: middle frontal gyrus) using a single sciMETv2.SL preparation...”*

- I like having a Figure that summarises the approach (Figure 6), but I find it hard to follow with all the UMAPs, arrows and colour schemes. Could the authors simplify the figure and make it more clear to the reader?

**We thank the reviewer for pointing this out. We now break the figure into two panels to convey the two major points. Panel a conveys the increased cluster resolution that can be obtained when incorporating capture, and panel b conveys that sizable single-cell methylome datasets can be generated without an excessive sequencing budget, making it more accessible to the broader research community. We hope this makes the message of the summary figure clearer.**

| **- A significant part of the results involve computational work. The authors should make the code accessible for everyone to reproduce the main results shown in the manuscript.**  **We agree completely. We now include an Additional Data File 4 which contains all code used for this study in archival form and now also refer to the actively developed GitHub repository.**  **Methods: *“Exact scripts used for data processing can be found in Additional File 4. These scripts are also available on GitHub (***[***https://github.com/adeylab/sciMETv2***](https://github.com/adeylab/sciMETv2)***) in an actively developed repository and may include future data processing improvements for processing sciMET and sciMET-cap datasets.”*** |  |
| --- | --- |

**Second round of review**

**Reviewer 1**

The authors have successfully addressed all my comments

**Reviewer 2**

I think the authors have responded to the reviewer’s comments thoughtfully and adequately. The additional analysis reported in Supplementary Figures 1 & 2 showing that capture does not introduce strand biases or skew recovery based on methylation status is very useful.

And the further clarification about how enrichment metrics in relation to ‘shadow coverage’ is also appreciated.

**Reviewer 3**

I thank the authors for addressing all my comments and I congratulate them for an excellent manuscript.
